# *Tert*-butyl benzoquinone: mechanism of biofilm eradication and potential for use as a topical antibiofilm agent

**DOI:** 10.1093/jac/dkw100

**Published:** 2016-04-27

**Authors:** N. Ooi, E. A. Eady, J. H. Cove, A. J. O'Neill

**Affiliations:** 1 Antimicrobial Research Centre and School of Molecular and Cellular Biology, University of Leeds, Leeds, UK; 2 Harrogate and District NHS Foundation Trust, Harrogate, UK

## Abstract

**Objectives:**

*Tert*-butyl benzoquinone (TBBQ) is the oxidation product of *tert*-butyl hydroquinone (TBHQ), an antimicrobial food additive with >40 years of safe use. TBBQ displays potent activity against *Staphylococcus aureus* biofilms *in vitro.* Here, we report on studies to further explore the action of TBBQ on staphylococcal biofilms, and provide a preliminary preclinical assessment of its potential for use as a topical treatment for staphylococcal infections involving a biofilm component.

**Methods:**

The antibacterial properties of TBBQ were assessed against staphylococci growing in planktonic culture and as biofilms in the Calgary Biofilm Device. Established assays were employed to measure the effects of TBBQ on biofilm structure and bacterial membranes, and to assess resistance potential. A living-skin equivalent was used to evaluate the effects of TBBQ on human skin.

**Results:**

TBBQ eradicated biofilms of *S. aureus* and other staphylococcal species at concentrations ≤64 mg/L. In contrast to other redox-active agents exhibiting activity against biofilms, TBBQ did not cause substantial destructuring of the biofilm matrix; instead, the antibiofilm activity of the compound was attributed to its ability to kill slow- and non-growing cells via membrane perturbation. TBBQ acted synergistically with gentamicin, did not damage a living-skin equivalent following topical application and exhibited low resistance potential.

**Conclusions:**

The ability of TBBQ to eradicate biofilms appears to result from its ability to kill bacteria regardless of growth state. Preliminary evaluation suggests that TBBQ represents a promising candidate for development as a topical antibiofilm agent.

## Introduction

Biofilms comprise structured communities of microorganisms in a self-produced extracellular matrix, usually attached to an organic or abiotic surface.^1^ For many bacteria, including a substantial proportion of those that cause human disease, the biofilm represents the usual mode of growth.^2^ Infections involving a substantial biofilm component (e.g. chronic wounds) are notoriously difficult to treat; not only does the physiological status of the bacteria inside the biofilm render them refractory to killing by extant antibacterial drugs, but the extracellular matrix acts to physically shield the inhabitants from attack by the host's immune system.^3^ One approach to address the current difficulties we face in treating biofilm infections is to discover new antibacterial agents that demonstrate substantial killing and/or eradication of bacterial biofilms.^4^

Here, we present a detailed characterization of one such candidate compound—*tert*-butyl benzoquinone (TBBQ). TBBQ is the spontaneous oxidation product of *tert*-butyl hydroquinone (TBHQ), a food preservative with over 40 years' safe use,^5^ and represents the chemical species responsible for the antibacterial activity previously ascribed to TBHQ.^6^ During a recent study to investigate the antibacterial properties of TBBQ, we were intrigued to find that this compound was able to eradicate preformed biofilms of the laboratory strain *Staphylococcus aureus* SH1000.^6^ We have therefore undertaken a more comprehensive investigation into the activity and mode of action of TBBQ on staphylococcal biofilms and conducted a preliminary assessment of its potential for use as a topical treatment for staphylococcal infections involving a biofilm component.

## Materials and methods

### General aspects

A panel of coagulase-positive and -negative staphylococci (*S. aureus* SH1000,^7,8^ *S. aureus* Mu50, *S. aureus* Oxford, *S. aureus* MRSA252, *S. aureus* USA300 FPR3757, *S. aureus* UAMS-1, *Staphylococcus epidermidis* RP62A*, Staphylococcus hominis* NR5871, *Staphylococcus haemolyticus* NCTC 11042, *Staphylococcus capitis* NCTC 11045 and *Staphylococcus lugdunensis* 31440) was employed for evaluating the antibacterial activity of TBBQ. Bacteria were cultured using Mueller–Hinton broth (MHB) and agar (Oxoid, Cambridge, UK), supplemented with calcium (50 mg/L, in the form of CaCl_2_) for studies involving daptomycin. Chemicals were obtained from Sigma–Aldrich (Poole, UK), unless otherwise stated.

### Evaluation of antibacterial activity

MICs were determined according to CLSI guidelines,^9^ whilst time–kill experiments were performed using exponential-phase, stationary-phase and persister cells, as previously described.^4^ Minimum biofilm eradication concentrations (MBECs) were determined using the Calgary Biofilm Device (CBD).^10^ Synergistic interactions between TBBQ and established antibacterial drugs were examined against biofilms grown on the CBD using the chequerboard method.^11^

### Mode-of-action studies

The effect of TBBQ on bacterial membrane potential was evaluated using the fluorescent dye 3,3′-dipropylthiadicarbocyanine iodide [DiSC_3_(5)] (Invitrogen, Paisley, UK), whilst physical membrane integrity was assessed by measuring leakage of potassium ions from staphylococci resuspended in HEPES-glucose buffer (5 mM, pH 7.2).^12^ The impact of TBBQ on biofilm structure was assessed by quantifying matrix material and adherent cells by staining with SYPRO^®^Ruby and SYTO^®^9 stains (Invitrogen), respectively.^4^

### Preliminary evaluation of potential for use of TBBQ as a topical antibiofilm agent

The effect of compounds on a human living-skin equivalent was assessed using fully differentiated, 28-day-old LabSkin™ (Innovenn, York, UK), as described previously.^4^ The potential for development of resistance to TBBQ was investigated by plating saturated bacterial cultures onto Mueller–Hinton agar containing TBBQ at 4 × MIC^13^ and using the extended-gradient MIC method of serial passage.^14^

## Results and discussion

We have previously demonstrated that TBBQ exhibits good antibacterial activity (MIC of 8 mg/L for the laboratory strain *S. aureus* SH1000) and sterilizes preformed biofilms of this same strain at 8 × MIC (MBEC of 64 mg/L).^6^ Here, we further evaluated the activity of TBBQ against planktonic and biofilm cultures of *S. aureus* clinical isolates (including MRSA and vancomycin-intermediate *S. aureus* strains) and other staphylococci capable of causing human disease. TBBQ inhibited bacterial growth and eradicated biofilms of all isolates (MIC 4–8 mg/L, MBEC 4–64 mg/L), with a potency equivalent to, or better than, that displayed against *S. aureus* SH1000, and at concentrations potentially achievable in skin via topical delivery.

**Figure 1.**
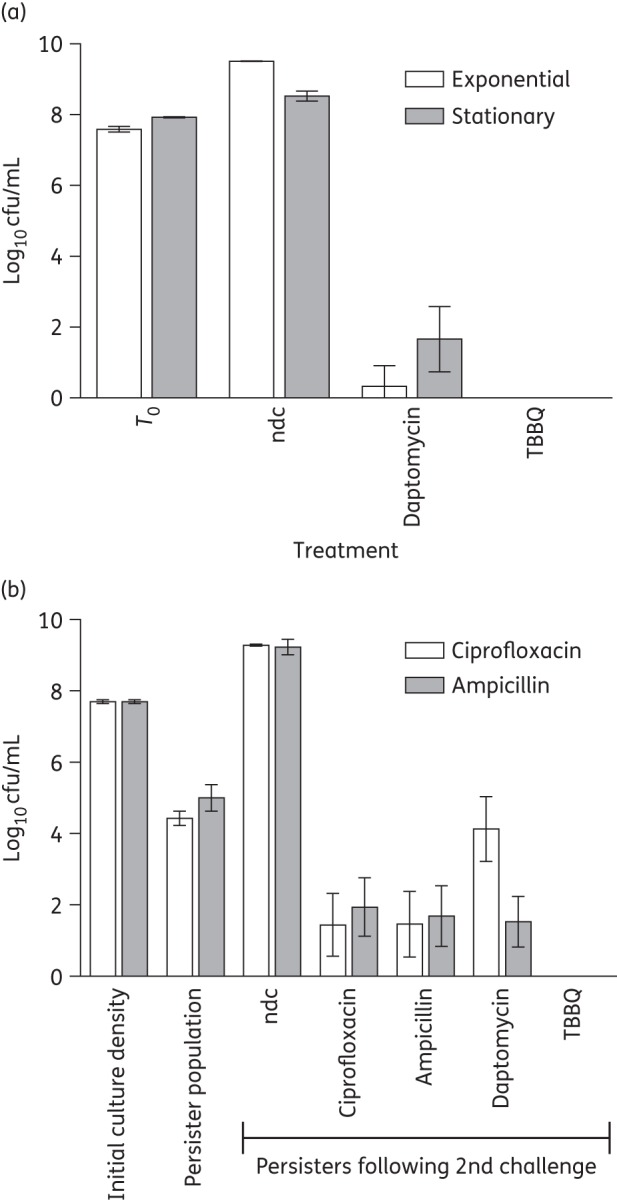

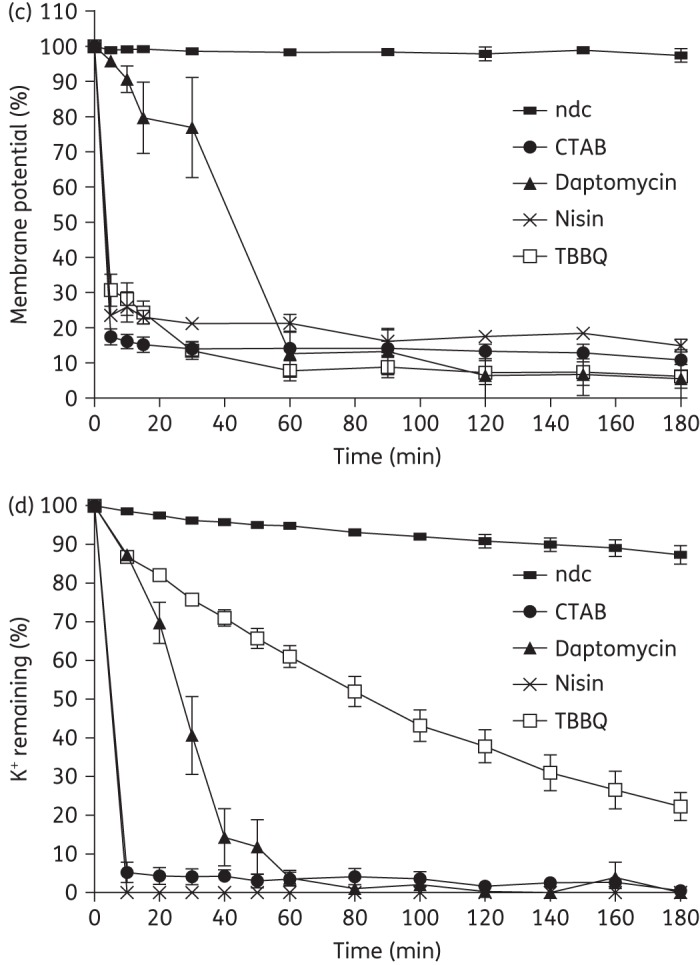
Antibacterial properties of TBBQ and comparator agents against *S. aureus* SH1000. (a) Viability of staphylococci from exponential- and stationary-phase cultures following exposure to TBBQ and comparator compounds at 256 mg/L for 24 h. *T*_0_ shows bacterial cell density prior to the addition of compounds. (b) Viability of persister cells (recovered after challenge with either ampicillin or ciprofloxacin) following exposure to TBBQ and comparator compounds at 10 × MIC for 24 h. (c) Effect of TBBQ and comparator agents at 4 × MIC on bacterial membrane potential. (d) Effect of compounds at 4 × MIC on leakage of intracellular potassium. All datum points represent means of at least three independent determinations, and error bars show standard deviations. CTAB, cetyltrimethylammonium bromide; K^+^, potassium ion; ndc, no-drug control.

Several redox-active compounds capable of eradicating staphylococcal biofilms do so by destructuring the biofilm matrix.^4^ Although TBBQ is a redox-active agent, it did not cause a significant reduction in the quantity of adhered matrix material or cells when tested against SH1000 biofilms at 4 × MBEC (256 mg/L) (data not shown), a result indicating that this compound exerts its antibiofilm activity through a different mechanism. The failure of established antibacterial drugs to eradicate bacterial biofilms has been attributed to the inability of these agents to effectively kill the large proportion of slow- or non-growing (SONG) cells, including persisters, present in biofilms.^15^ To assess whether the antibiofilm activity of TBBQ might result from its ability to kill SONG bacteria, we evaluated TBBQ-mediated killing of *S. aureus* SH1000 during exponential growth, in stationary phase and in the persister state. At 4 × MBEC (256 mg/L), TBBQ sterilized cultures (limit of detection of 10 cfu/mL) of both actively growing and non-growing (stationary phase) bacteria, a property not shared by the comparator agent, daptomycin (Figure 1a). Similarly, TBBQ was the only agent tested that could sterilize a population of persister cells (Figure 1b) (limit of detection of 1 cfu/mL).

A common feature of compounds capable of eradicating preformed bacterial biofilms is that they act to perturb the bacterial membrane.^16,17^ In our previous study on TBBQ, we showed using a dye penetration assay that this compound caused a reduction (∼35%) in bacterial membrane integrity following 10 min of exposure,^6^ suggesting that the antibacterial mode of action of TBBQ does indeed involve membrane perturbation. To confirm and further define the action of TBBQ on the bacterial membrane, we challenged *S. aureus* SH1000 with TBBQ and monitored the effect on membrane potential [using the fluorescent dye DiSC_3_(5)] and on the physical integrity of the membrane (by quantifying leakage of intracellular potassium ions) over time. TBBQ caused more rapid dissipation of membrane potential than the antibiotic daptomycin, yielding a similar profile to that seen for the lantibiotic nisin (Figure 1c). TBBQ also caused physical damage to the staphylococcal membrane; however, whilst the comparator membrane-perturbing agents all achieved essentially complete leakage of intracellular potassium from bacteria in ≤60 min, leakage of potassium from TBBQ-treated cells was more gradual and remained incomplete after 180 min (Figure 1d). Thus, the action of TBBQ on bacterial membranes can be distinguished from that of other membrane-active antibacterial agents, in that near-complete loss of membrane potential is observed well before substantial loss of membrane integrity becomes evident.

We examined whether TBBQ demonstrates improved activity against staphylococcal biofilms when combined with established antibacterial drugs. No synergy was observed (fractional inhibitory concentration index of >0.5) with respect to biofilm eradication when TBBQ was individually combined with the antibacterial drugs ciprofloxacin, erythromycin, oxacillin and tetracycline. However, synergy was observed (fractional inhibitory concentration index of ≤0.28) when TBBQ was combined with gentamicin. Gentamicin is used as a topical cream at 0.1% (1000 mg/L) for the treatment of infected wounds^18^ and, at this concentration, is unable to eradicate established staphylococcal biofilms *in vitro* (data not shown). However, the combination of TBBQ (2 mg/L) with gentamicin (0.1%) achieved eradication *in vitro*, suggesting that co-application of these agents might prove effective for the topical treatment of infections involving a biofilm component.

**Figure 2.**
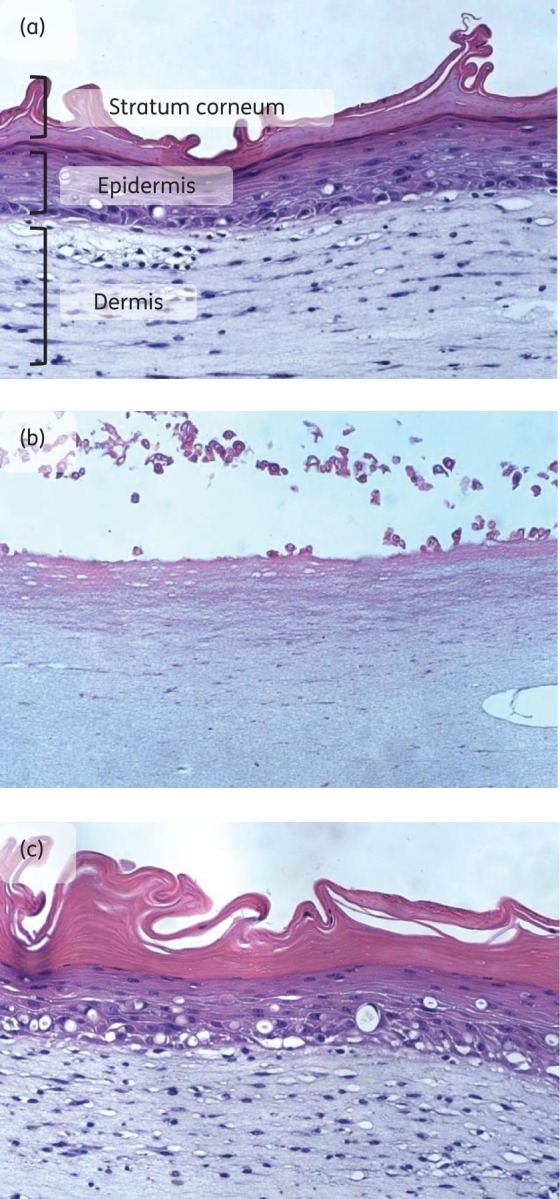
TBBQ does not cause visible damage to a living-skin equivalent. LabSkin™ was exposed to compounds for 24 h, and sections were subjected to haematoxylin and eosin staining. (a) Untreated control. (b) SDS (5% w/v). (c) TBBQ at 10 × MIC. This figure appears in colour in the online version of *JAC* and in black and white in the print version of *JAC*.

For a compound to be developed as a topical antibiofilm agent, it should not cause damage or significant irritation to human skin. *In vitro*, three-dimensional skin models (‘living-skin equivalents’), such as LabSkin™, represent an established means of evaluating the acute dermal toxicity of chemical compounds.^19^ Following exposure of LabSkin™ to TBBQ at 10 × MIC (80 mg/L) for 24 h, there was no increase in the release of the inflammatory cytokine IL-1α (data not shown). Furthermore, haematoxylin and eosin staining of tissue sections showed no visible detrimental effects following exposure of LabSkin™ to TBBQ (Figure 2c). Therefore, TBBQ does not physically damage or irritate fully differentiated human skin at concentrations above those required to eradicate staphylococcal biofilms. By contrast, exposure of LabSkin™ to the irritant SDS induced a 30-fold increase in release of IL-1α and was severely damaging to the skin structure, causing shedding of the stratum corneum and epidermis and injury to the dermis (Figure 2b).

Bacteria exposed to topical antibacterial agents may encounter high compound concentrations and thereby experience a strong selection pressure favouring the rapid development of resistance.^20^ Consequently, it is desirable for a candidate topical antibiofilm agent to exhibit low resistance potential. To evaluate the resistance potential of TBBQ, saturated cultures of *S. aureus* SH1000 were plated onto agar containing the compound at 4 × MIC; no resistant mutants were recovered (limit of detection, 5.0 × 10^−9^). We subsequently attempted to select TBBQ resistant mutants by extended serial passage in the presence of the compound. After 40 passages, a strain of SH1000 exhibiting a 4-fold increase in TBBQ MIC was recovered. By comparison, SH1000 subjected to 40 passages in the presence of daptomycin, an antibacterial drug usually considered to exhibit low resistance potential, resulted in a strain exhibiting a 16-fold increase in daptomycin MIC. Thus, TBBQ does not readily select substantial levels of resistance.

### Conclusions

The ability of TBBQ to eradicate biofilms appears to result from its membrane-perturbing activity, which allows it to kill bacteria regardless of growth state. TBBQ exhibits potent antibiofilm activity, an absence of detectable toxic effects on human skin and low resistance potential. This agent therefore represents a promising candidate for topical treatment, alone or in combination with gentamicin, of staphylococcal skin infections involving a biofilm component.

## Funding

This work was supported by BBSRC Industrial CASE studentship BB/G017158/1 in conjunction with Syntopix Group plc (known latterly as Evocutis plc).

## Transparency declarations

E. A. E. and J. H. C. are former employees of Syntopix Group plc (known latterly as Evocutis plc). J. H. C. owns shares in Evocutis plc. Both other authors: none to declare.
